# Supplementary material for: Dynalign II: common secondary structure prediction for RNA homologs with domain insertions
Source: Nucleic Acids Res. 2014 Nov 21;42(22):13939–48. doi: 10.1093/nar/gku1172 (PMC4267632; doi:10.1093/nar/gku1172)
Supplement: SUPPLEMENTARY DATA [file supp_gku1172_nar-02021-z-2014-File012.zip › manual/javadoc/overview-tree.html]

Class Hierarchy


JavaScript is disabled on your browser.


- Overview
- Package
- Class
- Tree
- Index
- Help

- Prev
- Next

- Frames
- No Frames

- All Classes

# Hierarchy For All Packages

Package Hierarchies:

- RNAstructure\_java\_drawing.source,
- RNAstructure\_java\_drawing.source.dialogs,
- RNAstructure\_java\_drawing.source.menus,
- RNAstructure\_java\_drawing.source.proxy,
- RNAstructure\_java\_drawing.source.runners,
- RNAstructure\_java\_interface.source,
- RNAstructure\_java\_interface.source.menus,
- RNAstructure\_java\_interface.source.startup,
- RNAstructure\_java\_interface.source.windows,
- RNAstructure\_java\_utilities

## Class Hierarchy

- java.lang.Object
  - RNAstructure\_java\_utilities.BorderBuilder (implements java.io.Serializable)
  - java.awt.Component (implements java.awt.image.ImageObserver, java.awt.MenuContainer, java.io.Serializable)
    - java.awt.Container
      - javax.swing.JComponent (implements java.io.Serializable)
        - javax.swing.AbstractButton (implements java.awt.ItemSelectable, javax.swing.SwingConstants)
          - javax.swing.JButton (implements javax.accessibility.Accessible)
            - RNAstructure\_java\_interface.source.windows.OligoWalkWindow.BarButton
          - javax.swing.JMenuItem (implements javax.accessibility.Accessible, javax.swing.MenuElement)
            - javax.swing.JMenu (implements javax.accessibility.Accessible, javax.swing.MenuElement)
              - RNAstructure\_java\_utilities.RNAstructureMenu (implements java.awt.event.ActionListener)
                - RNAstructure\_java\_drawing.source.menus.AnnotationMenu
                - RNAstructure\_java\_drawing.source.menus.DotsMenu
                - RNAstructure\_java\_drawing.source.dialogs.ImageDialog.PrintMenu
                - RNAstructure\_java\_drawing.source.menus.PlotMenu
                - RNAstructure\_java\_interface.source.menus.RolloverMenu
                  - RNAstructure\_java\_interface.source.menus.ConstraintsMenu
                  - RNAstructure\_java\_interface.source.menus.FileMenu
                  - RNAstructure\_java\_interface.source.menus.HelpMenu
                  - RNAstructure\_java\_interface.source.menus.NucleicAcidMenu
                  - RNAstructure\_java\_interface.source.windows.OligoWalkWindow.OligoWalkResultsMenu
                  - RNAstructure\_java\_interface.source.windows.SequenceDisplayWindow.EditMenu
                  - RNAstructure\_java\_interface.source.windows.SequenceDisplayWindow.ReadMenu
                - RNAstructure\_java\_drawing.source.menus.StructureMenu
          - javax.swing.JToggleButton (implements javax.accessibility.Accessible)
            - javax.swing.JCheckBox (implements javax.accessibility.Accessible)
              - RNAstructure\_java\_utilities.HTMLCheckBox
        - javax.swing.JComboBox<E> (implements javax.accessibility.Accessible, java.awt.event.ActionListener, java.awt.ItemSelectable, javax.swing.event.ListDataListener)
          - RNAstructure\_java\_interface.source.windows.OligoWalkWindow.StringComboBox
        - javax.swing.JInternalFrame (implements javax.accessibility.Accessible, javax.swing.RootPaneContainer, javax.swing.WindowConstants)
          - RNAstructure\_java\_interface.source.windows.InternalWindow (implements java.awt.event.ActionListener)
            - RNAstructure\_java\_interface.source.windows.DrawingWindow
            - RNAstructure\_java\_interface.source.windows.ModuleWindow
              - RNAstructure\_java\_interface.source.windows.DynalignFoldWindow
              - RNAstructure\_java\_interface.source.windows.DynalignRefoldWindow
              - RNAstructure\_java\_interface.source.windows.Efn2Window
              - RNAstructure\_java\_interface.source.windows.FoldDoubleWindow
              - RNAstructure\_java\_interface.source.windows.FoldSingleWindow
              - RNAstructure\_java\_interface.source.windows.FoldSuboptimalWindow
              - RNAstructure\_java\_interface.source.windows.MaxExpectWindow
              - RNAstructure\_java\_interface.source.windows.MultiWindow
                - RNAstructure\_java\_interface.source.windows.MultilignWindow
                - RNAstructure\_java\_interface.source.windows.TurboFoldWindow
              - RNAstructure\_java\_interface.source.windows.OligoScreenWindow
              - RNAstructure\_java\_interface.source.windows.OligoWalkWindow
              - RNAstructure\_java\_interface.source.windows.PartitionDoubleWindow
              - RNAstructure\_java\_interface.source.windows.PartitionSingleWindow
              - RNAstructure\_java\_interface.source.windows.ProbKnotWindow
              - RNAstructure\_java\_interface.source.windows.PseudoknotWindow
              - RNAstructure\_java\_interface.source.windows.RefoldWindow
              - RNAstructure\_java\_interface.source.windows.StochasticWindow
            - RNAstructure\_java\_interface.source.windows.SequenceDisplayWindow
        - javax.swing.JMenuBar (implements javax.accessibility.Accessible, javax.swing.MenuElement)
          - RNAstructure\_java\_interface.source.menus.DynamicMenuBar
        - javax.swing.JPanel (implements javax.accessibility.Accessible)
          - RNAstructure\_java\_utilities.BorderedButtonPanel
          - RNAstructure\_java\_utilities.FieldPanel
            - RNAstructure\_java\_utilities.FieldPanel.FilePanel
            - RNAstructure\_java\_utilities.FieldPanel.NumberPanel
          - RNAstructure\_java\_interface.source.windows.OligoWalkWindow.SpinnerPanel
          - RNAstructure\_java\_utilities.RadioButtonPanel
        - javax.swing.JScrollPane (implements javax.accessibility.Accessible, javax.swing.ScrollPaneConstants)
          - RNAstructure\_java\_utilities.ScrollerPane
        - javax.swing.text.JTextComponent (implements javax.accessibility.Accessible, javax.swing.Scrollable)
          - javax.swing.JTextField (implements javax.swing.SwingConstants)
            - RNAstructure\_java\_utilities.FileField
            - RNAstructure\_java\_utilities.NumberField
              - RNAstructure\_java\_utilities.NumberField.DoubleField
              - RNAstructure\_java\_utilities.NumberField.FloatField
              - RNAstructure\_java\_utilities.NumberField.IntegerField
      - java.awt.Window (implements javax.accessibility.Accessible)
        - java.awt.Dialog
          - javax.swing.JDialog (implements javax.accessibility.Accessible, javax.swing.RootPaneContainer, javax.swing.WindowConstants)
            - RNAstructure\_java\_drawing.source.dialogs.ImageDialog (implements java.awt.print.Printable)
              - RNAstructure\_java\_drawing.source.dialogs.PlotDialog
              - RNAstructure\_java\_drawing.source.dialogs.StructureDialog
            - RNAstructure\_java\_utilities.ValueSelectionDialog
              - RNAstructure\_java\_interface.source.menus.ConstraintsMenu.NucOrHelixDialog
              - RNAstructure\_java\_drawing.source.menus.DotsMenu.ColorDialog
              - RNAstructure\_java\_drawing.source.menus.DotsMenu.RangeDialog
              - RNAstructure\_java\_drawing.source.dialogs.ImageDialog.ZoomDialog
              - RNAstructure\_java\_interface.source.windows.OligoWalkWindow.OligoSelectionDialog
              - RNAstructure\_java\_drawing.source.menus.StructureMenu.MoveDialog
        - java.awt.Frame (implements java.awt.MenuContainer)
          - javax.swing.JFrame (implements javax.accessibility.Accessible, javax.swing.RootPaneContainer, javax.swing.WindowConstants)
            - RNAstructure\_java\_interface.source.startup.ApplicationRootFrame
  - RNAstructure\_java\_drawing.source.proxy.DotPlotBackend
  - RNAstructure\_java\_drawing.source.proxy.DotPlotBackendProxyJNI
  - RNAstructure\_java\_utilities.FileChooser (implements java.io.Serializable)
  - RNAstructure\_java\_utilities.ImageGrabber (implements java.io.Serializable)
  - RNAstructure\_java\_utilities.MacChecker (implements java.io.Serializable)
  - RNAstructure\_java\_interface.source.startup.RNAstructure (implements java.io.Serializable)
  - RNAstructure\_java\_interface.source.startup.RNAstructureBackendCalculator
  - RNAstructure\_java\_interface.source.startup.RNAstructureBackendCalculatorProxyJNI
  - RNAstructure\_java\_interface.source.menus.RolloverMenu.RolloverListener (implements java.awt.event.MouseListener, java.io.Serializable)
  - RNAstructure\_java\_drawing.source.runners.RunnerTemplate
    - RNAstructure\_java\_drawing.source.runners.PlotRunner (implements java.io.Serializable)
    - RNAstructure\_java\_drawing.source.runners.StructureRunner (implements java.io.Serializable)
  - RNAstructure\_java\_utilities.SimpleDialogHandler (implements java.io.Serializable)
  - RNAstructure\_java\_drawing.source.proxy.StructureBackend
  - RNAstructure\_java\_drawing.source.proxy.StructureBackendProxyJNI

## Enum Hierarchy

- java.lang.Object
  - java.lang.Enum<E> (implements java.lang.Comparable<T>, java.io.Serializable)
    - RNAstructure\_java\_utilities.FileChooser.Types

- Overview
- Package
- Class
- Tree
- Index
- Help

- Prev
- Next

- Frames
- No Frames

- All Classes
